# Supplementary material for: SIRT1 Activator E1231 Alleviates Nonalcoholic Fatty Liver Disease by Regulating Lipid Metabolism
Source: Curr Issues Mol Biol. 2023 Jun 8;45(6):5052–70. doi: 10.3390/cimb45060321 (PMC10297637; doi:10.3390/cimb45060321)
Supplement: Supplementary file 1 [file cimb-45-00321-s001.zip › cimb-2375051-supplementary.pdf]

SIRT1 activator E1231 alleviates nonalcoholic fatty liver disease by regulating lipid metabolism

Jiangxue Han[†, 1], Shunwang Li[†, 1], Weizhi Wang[1], Xinhai Jiang[1], Chao Liu[1], Lijuan Lei[1], Yining Li[1], Ren Sheng[1], Yuyan Zhang[1], Yexiang Wu[1], Jing Zhang[1], Yuhao Zhang[1], Yanni Xu[*,1], Shuyi Si[*,1]

[1]NHC Key Laboratory of Biotechnology of Antibiotics, National Center for Screening Novel Microbial Drugs, Institute of Medicinal Biotechnology, Chinese Academy of Medical Sciences & Peking Union Medical College (CAMS & PUMC), Tiantan Xili 1#, Beijing 100050, China.

*Correspondence: xuyanni2010@imb.pumc.edu.cn (Y.X.); sisy@imb.pumc.edu.cn (S.S.)

†These authors contributed equally to this work.

1. Materials and Methods

1.1 RNA isolation and real-time quantitative polymerase chain reaction PCR (RT-qPCR)

Total RNA from liver tissues were extracted with TRIzol™ reagent (1559602, Invitrogen, CA, USA) and reverse-transcribed to cDNA using the PerfectStart Uni RT&qPCR Kit (AUQ01, TransGen, Beijing, China). The RT-qPCR assays were performed using PerfectStart Green qPCR SuperMix (AQ601, TransGen, Beijing, China) on a FTC3000 RT-qPCR system (Funglyn Biotech Inc, Toronto, Canada). The mRNA expression levels of the studied genes were calculated relative to *Gapdh*. The primers were listed in Table S1.

Table S1. The primer sequences of the target genes.

Gene	Forward Primer	Reverse Primer
Il6	TAGTCCTTCCTACCCCAATTTCC	TTGGTCCTTAGCCACTCCTTC
Tnfα	CCCTCACACTCAGATCATCTTCT	GCTACGACGTGGGCTACAG
Il1β	GCAACTGTTCCTGAACTCAACT	ATCTTTTGGGGTCCGTCAACT
Gapdh	AGGTCGGTGTGAACGGATTTG	TGTAGACCATGTAGTTGAGGTCA
Pparγ1	GAGTGTGACGACAAGATTTG	GGTGGGCCAGAATGGCATCT
Pparγ2	TCTGGGAGATTCTCCTGTTGA	GGTGGGCCAGAATGGCATCT
Adrp	GACCTTGTGTCCTCCGCTTAT	CAACCGCAATTTGTGGCTC
Cd36	AATGGCACAGACGCAGCCT	GGTTGTCTGGATTCTGGA
Srebp1c	GGAGCCATGGATTGCACATT	CCTGTCTCACCCCCAGCATA
Fas	GCTGCGGAAACTTCAGGAAAT	AGAGACGTGTCACTCCTGGACTT
Scd1	CCCCTGCGGATCTTCCTTAT	AGGGTCGGCGTGTGTTTCT
Acc	GGACAGACTGATCGCAGAGAAAG	TGGAGAGCCCCACACACA

1.2 Malondialdehyde (MDA) assay

The liver malondialdehyde (MDA) contents were measured with a liquid sample MDA assay kit (#E2019, APPLYGEN, Beijing, China). Briefly, the liver tissue was weighed and homogenized in the buffer from the MAD assay kit at 4 °C. The homogenates were centrifuged (4 °C, 10000 g) for 10 min. The supernatants were collected for MDA concentration (absorption wavelength: 535 nm) and protein concentration (absorption wavelength: 562 nm) analysis according to the manufacturer's protocols using a Multimode Plate Reader (EnVision 2105, PerkinElmer, Fremont, CA).

2. Results

2.1 E1231 treatment inhibited liver inflammation in a high-fat and high-cholesterol diet (HFHC)-induced NAFLD mice

The effects of E1231 on inflammation were examined. RT-qPCR results showed that the mRNA levels of inflammatory cytokines including tumor necrosis factor α (*Tnfα*) ($p < 0.05$), interleukin-1β (*Il1β*) ($p = 0.12$), and interleukin-6 (*Il6*) ($p = 0.059$) in the livers of HFHC group were higher than those in the CD group (Figure S1). E1231 treatment decreased the mRNA levels of *Tnfα* ($p = 0.065$), *Il1β* ($p = 0.14$), and *Il6* ($p = 0.068$) compared with the HFHC group (Figure S1). These data indicated that E1231 treatment could inhibit inflammation *in vivo*.

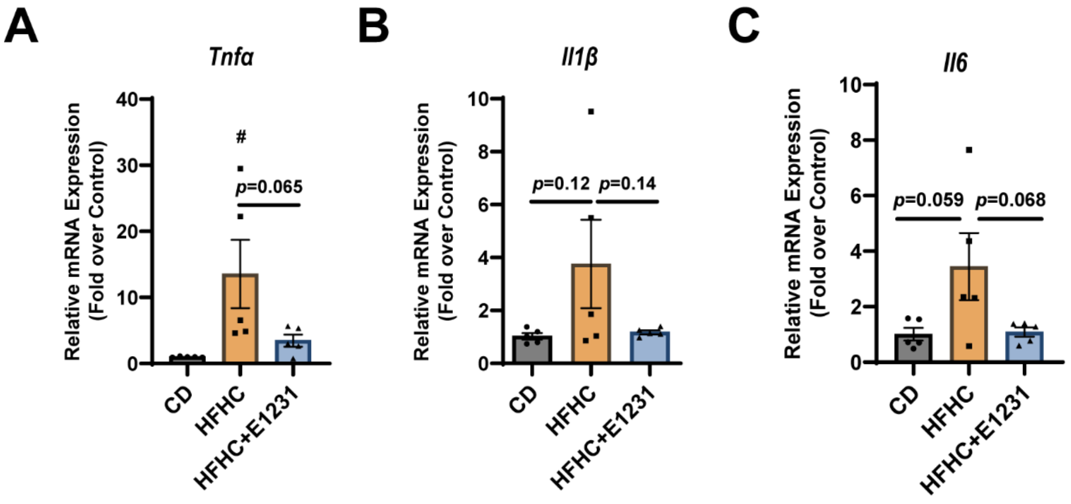

Figure S1. Effects of E1231 on liver inflammatory cytokines in NAFLD mice. (A-C) The mRNA level of inflammatory factors *Tnfα* (A), *Il1β* (B) and *Il6* (C) in the liver of a high-fat and high-cholesterol diet (HFHC) induced NAFLD mice. n = 5 per group. The data are presented as the mean ± SEM. One-way ANOVA was used for analysis. HFHC group vs. CD group: #$p < 0.05$.

2.2 E1231 treatment decreased MDA in HFHC-induced NAFLD mice

Chronic impairment of lipid metabolism leads to alterations of the oxidant/antioxidant balance, cellular lipotoxicity, lipid peroxidation, chronic endoplasmic reticulum (ER) stress, and mitochondrial dysfunction. MDA is a marker of oxidative stress-induced lipid peroxidation product. As shown in Figure S2, HFHC feeding significantly increased the liver MDA contents compared with the CD feeding group, whereas E1231 treatment obviously decreased the liver MDA contents compared with HFHC group (Figure S2). These data indicated that E1231 treatment could improve chronic lipid oversupply induced oxidative stress and lipid peroxidation.

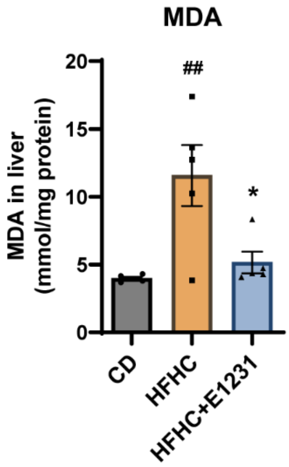

Figure S2. E1231 decreased malondialdehyde (MDA) contents in NAFLD mice. The MDA level in the livers of HFHC induced NAFLD mice treated with or without E1231. n = 5 per group. The data are presented as the mean ± SEM. One-way ANOVA was used for analysis. HFHC group vs. CD group: ##$p < 0.01$, * $p < 0.05$.
